# The Impact of Post-COVID-19 Syndrome in Adolescents: A Pilot Study

**DOI:** 10.7759/cureus.40655

**Published:** 2023-06-19

**Authors:** Jane El Khoury, May Skoury, Marc Y El Khoury

**Affiliations:** 1 High School, Harrison High School, Harrison, USA; 2 Nursing, Harrison Central School District, Harrison, USA; 3 Department of Medicine, Division of Infectious Diseases, New York Medical College, Valhalla, USA

**Keywords:** covid-19, sars-cov-2, school performance, long hauler, post-acute covid-19

## Abstract

Background: Post-COVID-19 syndrome has emerged as a long-term complication in adults and children; its effect on adolescents’ performance in school is not well studied.

Objectives: To study the physical/psychological impact of prolonged post-COVID-19 symptoms on school performance.

Methods: This is a cross-sectional study using Google Forms, a web-based fully anonymized survey of children in grades 10-12.

Results: The study included 54 students with a mean age of 16 years of whom 32 had COVID-19. Two were hospitalized and 10 had symptoms lasting more than four weeks. Commonly reported chronic symptoms were fatigue and cough. Seven students quit sports; eight had a decrease in their academic performance. Adolescents being infected more than once or not being fully vaccinated were more likely to develop prolonged symptoms and quit sports while academic performance in school was not affected. Three out of 10 (30%) students who had COVID-19 and responded to the questionnaire reported not seeking help.

Conclusion: Post-COVID-19 syndrome is associated with a decline in physical but not mental performance in school. Being infected more than once with SARS-CoV-2 seems to play an important role in the persistence of post-COVID-19 symptoms despite the fact that some adolescents are hesitant to seek medical or psychological care.

## Introduction

Coronavirus disease 2019 (COVID-19), has been associated with significant worldwide morbidity and mortality [1]. The understanding of the long-term effects of COVID-19 (post-acute COVID-19 syndrome) is still evolving. The residual effects of severe acute respiratory syndrome coronavirus 2 (SARS-CoV-2) infection include fatigue, dyspnea, chest pain, cognitive disturbances, and arthralgia [2]. A study of 183 adults in the United States [3], showed that fatigue, dyspnea, and psychological distress, were present 35 days post-discharge in approximately 30% of patients. The severity of the acute illness in that study was shown to be associated with the persistence of symptoms [2]. The definition of a long hauler or a person suffering from post-acute COVID-19 has been suggested to include the persistence of symptoms beyond three or four weeks from the initial infection [4]. Until recently, published data on the prolonged effect of COVID-19 in children has been limited to multisystem inflammatory syndrome [5]. Persistence of post-infectious signs and symptoms (loss of taste or smell, cough, hair loss, chest pain, abnormal liver enzymes, skin rashes, fatigue and malaise, fever and chills, diarrhea, cardiorespiratory signs and symptoms, myositis, myocarditis, anxiety and requiring mental health therapy) beyond four weeks in children has been reported [6], however, the impact on school performance was not addressed. Our pilot study’s main objective was to look at the physical/psychological impact of post-acute COVID-19 on children’s school performance. Secondary objectives included looking for the correlation between prolonged symptoms (fatigue, dyspnea, chest pain, cognitive disturbances, and arthralgia) and age, race, blood type, SARS-CoV-2 variant, the severity of initial infection, first or second infection and presence of a family history of prolonged symptoms.

## Materials and methods

Our cross-sectional pilot study was designed to look at the physical and psychological impact of COVID-19 on children’s school performance at a high school in Lebanon. The inclusion criteria included children from grades (10-12) attending high school. Exclusion criteria included children with mental disabilities precluding them from accurately answering the questionnaire. The survey questionnaire and protocol were approved by the New York Medical College General Medical and Behavioral Institutional Review Board (IRB) with approval number 15182. The survey was designed as a web-based (fully anonymized) survey using Google Forms (Google Inc, 2021) to ease distribution and completion. In cooperation with the school’s administration, the survey link was shared with the students with the help of their teachers. We also requested the teachers to remind the students periodically to complete the survey. Participation was voluntary, and no incentives were provided to the participants. The fully anonymized data was obtained from Google Forms at the end of the survey time and stored safely in a password-protected file for further analysis. Statistical analysis was done using SPSS software version 27 (IBM Corp., Armonk, NY). Mean and standard deviations for all continuous variables, as well as, percentages for categorical variables were calculated. Chi-square testing was done to find if there is a significant relationship between different factors and post-acute COVID-19 and if the impact of COVID-19 in those who developed chronic symptoms was significantly different from those who did not have chronic symptoms. This article was previously posted to the medRxiv preprint server on April 27, 2020.

## Results

We surveyed 54 students, 15 to 18 years old, 32 females, 45 Caucasians, nine belonging to minority groups (Asian, Black, and Hispanic). Six students smoked/vaped. Nine students were not fully vaccinated (no vaccination or only one dose of an mRNA vaccine). Thirty-two (59.3%) reported having had COVID-19, and 7/32 (21.8%) were infected more than once. Twenty-one had COVID-19 between December 2021 and May 2022 during the surge of the Omicron variant (BA1), and 12 between April 2020 and May 2021. Two (6.2%) students were hospitalized. Fourteen students had COVID-19 despite being fully vaccinated. The most common acute symptoms were fatigue, headaches, and fever.

While many reported a short illness, 10 (31.3%) students reported symptoms for more than four weeks (two students from six to nine months). The symptoms lasting over four weeks included fatigue or cough in 30%, dyspnea or depression/anxiety in 20%, difficulty concentrating, headaches, loss of smell or taste, and myalgia/arthralgia in 10%. Half of the children with symptoms lasting more than four weeks (long haulers) had symptoms persisting after the first COVID-19, while the other half had prolonged symptoms after subsequent infections. Six out of 38 (15.8%) students reported that one of their family members was a long hauler. Seven out of 30 (23.3%) quit sports for the season. Eight out of 30 (26.6%) had a decline in their academic performance with worsening grades following COVID-19. Of the 10 students with prolonged symptoms, 60% were fully vaccinated and 60% had COVID-19 during the Omicron surge. Factors found to be associated with prolonged symptoms included having had COVID-19 more than once with a trend towards significance for not being fully vaccinated. Long haulers were found to be more likely to quit sports, however, their school grades were not found to be significantly affected compared to students without post-acute COVID-19 syndrome (Table 1).

**Table 1:** Crosstabulation table of demographic, clinical and laboratory factors potentially associated with prolonged symptoms COVID-19: Coronavirus disease 2019; SARS-CoV-2: Severe acute respiratory syndrome coronavirus 2; NS: Non-significant **No vaccine or only one dose of either Moderna or Pfizer mRNA vaccine

	Students with symptoms lasting >4 weeks/Respondents (N=10)	Students with symptoms lasting <4 weeks/Respondents (N=22)	p-value
Sex (Female)	6	15	0.65 (NS)
Race (not White)	2	4	0.68 (NS)
Smoking or vaping	1	1	0.55 (NS)
Not fully vaccinated for SARS-CoV-2 **	8	9	0.07 (NS)
Having COVID-19 more than once	5	2	0.031
Having a sibling who is a long hauler	3	3	0.2 (NS)
Quitting sport	5	2	0.028
Grades declined	4	4	0.3 (NS)
SARS-CoV-2 Alpha variant	3	7	0.60 (NS)
Blood Type B or AB	3	2	0.18 (NS)

**Figure 1:**
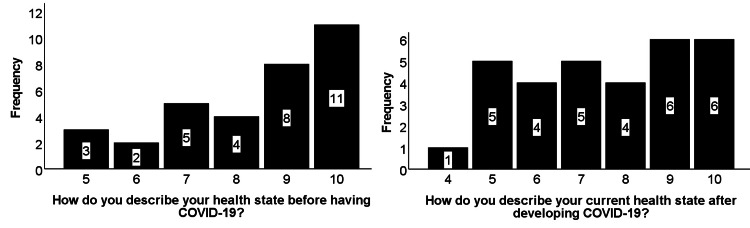
Self-health assessment before and after COVID-19 Self-health assessment before and after COVID-19 using a visual score from 1 to 10, with 10 being in the best physical condition and 1 being in the worst shape. The Y-axis represents the number of respondents, and the X-axis represents the visual score. The bar graph shows a decrease in post-COVID-19 health scores with an increase of almost 50% in children with scores 5 and 6 and an almost 50% decrease in the number of children with a score of 10.

Using a visual scale (from 1 to 10) to describe their health, many students who had COVID-19 reported some form of deterioration of their perceived general health status as seen in Figure 1.

## Discussion

Our study is a pilot study based on a survey using a Google form to reach a large number of students in a short period of time while maintaining anonymity. This study involves a specific age and setting that is understudied. In a previous survey by the world health organization, the COVID-19 pandemic was noted to have a significant impact on school-aged children's mental health and their performance in school in a general sense [7]. The impact of the infection itself and other associated factors was not studied. In our study, 16% of students were unvaccinated. While being unvaccinated was not statistically associated with developing prolonged symptoms that lasts more than four weeks, there was a trend toward significance (p = 0.07). This is likely related to the small sample size of this study. Having COVID-19 more than once was significantly associated with prolonged symptoms. The underlying pathophysiology of this finding remains unclear and warrants further study. There was no association between having a long hauler family member and an increased risk of developing prolonged symptoms making it less likely that an underlying hereditary trait shared among certain family members could be playing a role. Unlike the study by Raos et al. that showed a correlation between outcome and admission to the intensive care unit during the acute illness phase and post-acute COVID-19, our study did not [6]. Other studies also showed a correlation between outcome and race [8], blood type [9], and infecting variant type [10], while ours did not and this is likely due to the small number of studied subjects.

Despite the fact that the COVID-19 pandemic affected the well-being of many children whether infected or not, the effect on those who develop prolonged symptoms seems to be far greater than the other groups. More kids in this group quit sports for the season, however, there was no decline in their academic grades compared to their peers, which warrants special attention on the physical impact of this complication. Race or sex or smoking/vaping were not shown to have a significant effect in this age group. In this study, 3/10 (30%) of participants who had COVID-19 (10 out of 32 responded to this question) have not sought medical attention which should not be taken lightly. The most common prolonged symptoms reported were fatigue, cough, and central nervous system/mental disorder. Parents, teachers, and healthcare providers should be aware of the significant impact of post-acute COVID-19 on children with prolonged symptoms, warranting early screening and intervention in a timely manner to maintain their kids’ well-being.

## Conclusions

Having had recurrent COVID-19 infections appears to be associated with post-acute COVID-19 syndrome. Not being fully vaccinated against SARS-CoV-2 did not appear to fully play a protective role (only trends toward significance) possibly due to the emergence of the Omicron variant. Adolescents whose symptoms last more than four weeks are most affected by an increased risk of decline in their physical well-being and performance in sports at school.

Many adolescents do not seek help for ongoing symptoms whether physical or psychological. A larger study involving several school districts is necessary to evaluate the magnitude of this syndrome which helps better allocate appropriate resources to most affected districts.
